# Integrating 5-Hydroxymethylcytosine into the Epigenomic Landscape of Human Embryonic Stem Cells

**DOI:** 10.1371/journal.pgen.1002154

**Published:** 2011-06-23

**Authors:** Keith E. Szulwach, Xuekun Li, Yujing Li, Chun-Xiao Song, Ji Woong Han, SangSung Kim, Sandeep Namburi, Karen Hermetz, Julie J. Kim, M. Katharine Rudd, Young-Sup Yoon, Bing Ren, Chuan He, Peng Jin

**Affiliations:** 1 Department of Human Genetics, Emory University School of Medicine, Atlanta, Georgia, United States of America; 2 Department of Chemistry and Institute for Biophysical Dynamics, The University of Chicago, Chicago, Illinois, United States of America; 3 Division of Cardiology, Department of Medicine, Emory University School of Medicine, Atlanta, Georgia, United States of America; 4 Ludwig Institute for Cancer Research, La Jolla, California, United States of America; 5 Department of Cellular and Molecular Medicine, Institute of Genomic Medicine, University of California San Diego, La Jolla, California, United States of America; 6 UCSD Moores Cancer Center, University of California San Diego, La Jolla, California, United States of America; The Hospital for Sick Children and University of Toronto, Canada

## Abstract

Covalent modification of DNA distinguishes cellular identities and is crucial for regulating the pluripotency and differentiation of embryonic stem (ES) cells. The recent demonstration that 5-methylcytosine (5-mC) may be further modified to 5-hydroxymethylcytosine (5-hmC) in ES cells has revealed a novel regulatory paradigm to modulate the epigenetic landscape of pluripotency. To understand the role of 5-hmC in the epigenomic landscape of pluripotent cells, here we profile the genome-wide 5-hmC distribution and correlate it with the genomic profiles of 11 diverse histone modifications and six transcription factors in human ES cells. By integrating genomic 5-hmC signals with maps of histone enrichment, we link particular pluripotency-associated chromatin contexts with 5-hmC. Intriguingly, through additional correlations with defined chromatin signatures at promoter and enhancer subtypes, we show distinct enrichment of 5-hmC at enhancers marked with H3K4me1 and H3K27ac. These results suggest potential role(s) for 5-hmC in the regulation of specific promoters and enhancers. In addition, our results provide a detailed epigenomic map of 5-hmC from which to pursue future functional studies on the diverse regulatory roles associated with 5-hmC.

## Author Summary

Recent studies revealed the oxygenase-catalyzed production of 5-hydroxymethylcytosine (5-hmC) as a modification to mammalian DNA. 5-hmC is known to play important roles in self-renewal and cell lineage specification in embryonic stem (ES) cells, suggesting a potential role for 5-hmC–mediated epigenetic regulation in modulating the pluripotency of ES cells. To unveil this new regulatory paradigm in human ES cells, here we use a 5-hmC–specific chemical labeling approach to capture 5-hmC and profile its genome-wide distribution in human ES cells. We show that 5-hmC is an important epigenetic modification associated with the pluripotent state that could play role(s) in a subset of promoters and enhancers with defined chromatin signatures in ES cells.

## Introduction

The potency and fate of a cell can be influenced strongly by the covalent modification of cytosine methylation at carbon five. This critical epigenetic mark influences cellular potency and differentiation by modulating DNA-protein interactions, which direct epigenomic states and transcriptional processes, allowing otherwise common genomes to be expressed as distinct cell types. DNA-methylation-mediated epigenomic processes include dosage compensation, control over aberrant retrotransposon expression, and regulation of centromeric and telomeric heterochromatin [1]. The importance of such processes is exemplified by the essential requirement for DNA methyltransferases (DNMT1, DNMT3A, and DNMT3B) in embryonic and early mammalian development [2], [3].

Coincident with critical roles for DNA methyltransferases in the regulation of pluripotency, Fe(II)/α-ketoglutarate-dependent hydroxylation of 5-mC to 5-hydroxymethylcytosine (5-hmC) by Ten-eleven translocation (Tet) family proteins also contributes to the maintenance of pluripotency [4]–[6]. Discovery of this new epigenetic modification raises the possibility that 5-hmC could alter chromatin structure and thereby contribute to gene regulation. Recent functional studies have shown that Tet proteins, particularly Tet1 and Tet2, are required for ES cell self-renewal and maintenance. However, despite the emergence of these important roles for Tet family proteins, and therefore 5-hmC-associated regulation in ES cells, the genomic- and chromatin-associated contexts of 5-hmC have gone unexplored in human embryonic stem cells.

Although there are detailed chromatin state maps of histone modifications in human embryonic stem cells, much less is known about the distinction between 5-hmC and 5-mC localization, largely because of the inability of bisulfite sequencing to resolve the two marks [7], [8]. Recent studies indicate distinct differences in the presence of stable 5-hmC and Tet1 in mouse ES cells, where strong promoter-proximal Tet1 binding is inversely correlated with the presence of both 5-mC and 5-hmC [9]–[13], providing putative support for a Tet1-associated demethylation mechanism in the maintenance of unmethylated active promoters. Interestingly, these studies indicate that while Tet1 binding sites are highly enriched at transcription start sites (TSSs) in mouse ES cells, a significant fraction of detectable 5-hmC lies within gene bodies and other regulatory regions, which is also consistent with our previous study mapping 5-hmC genome-wide in mouse cerebellum [14]. Furthermore, at regions bound by both Polycomb (PRC2) and Tet1, the presence of 5-hmC is associated with a repressive state, indicating diverse regulatory roles for 5-hmC that depend at least in part on its chromatin context. Whether localization of 5-hmC with other distinct chromatin signatures results in diverse regulatory mechanisms remains to be explored.

To unravel the biology of 5-hmC, we recently developed a selective chemical labeling method for 5-hmC by using T4 bacteriophage ß-glucosyltransferase to transfer an engineered glucose moiety containing an azide group onto the hydroxyl group of 5-hmC, which in turn can chemically incorporate a biotin group for detection, affinity enrichment, and sequencing. Here, to understand the role of 5-hmC in the epigenomic landscape of pluripotent cells, we profiled the genome-wide 5-hmC distribution and correlated it with the genomic profiles of 11 diverse histone modifications and six transcription factors in human ES cells. By integrating genomic 5-hmC signals with maps of histone enrichment, we link particular pluripotency-associated chromatin contexts with 5-hmC. Intriguingly, through additional correlations with defined chromatin signatures at promoter and enhancer subtypes, we found distinct enrichment of 5-hmC at enhancers marked with H3K4me1 and H3K27ac. These results suggest potential role(s) for 5-hmC in the regulation of specific promoters and enhancers. In addition, our results provide a detailed epigenomic map of 5-hmC from which to pursue future functional studies on the diverse regulatory roles associated with 5-hmC.

## Results

### Exclusion of 5-hmC from metaphase pericentromeric heterochromatin

**Figure 1:**
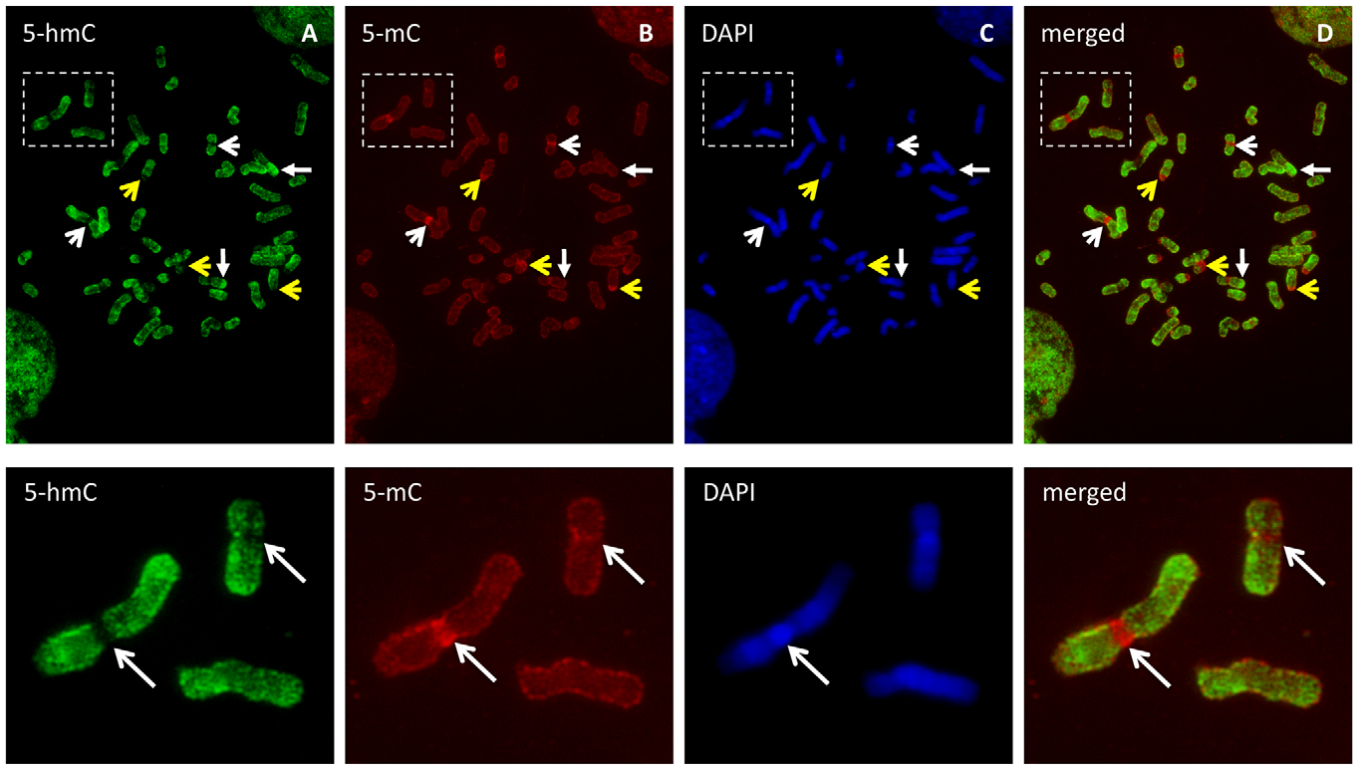
5-hmC- and 5-mC-specific immunostaining of metaphase chromosomes from human ES cells. Metaphase chromosomes from human embryonic stem cells were immunostained with antibodies specific to 5-hmC and 5-mC. A–D, Both 5-hmC and 5-mC could be observed along the chromosome arms. Strong 5-mC signal (yellow open arrow) but distinct exclusion of 5-hmC (white open arrow) from the heterochromatin of pericentromeres and the Y chromosome was observed. 5-hmC is enriched in some regions of multiple chromosomes (solid arrow). E–H, Representative images showing 5-hmC is strongly depleted from 5-mC-enriched pericentromeric region of chromosome 1 as indicated by arrow.

To assess the distribution and general chromatin context of 5-hmC in human embryonic stem (ES) cells, we first evaluated the cytogenetic localization of both 5-mC and 5-hmC by immunostaining metaphase chromosomes of human ES cells (Figure S1). Both 5-mC and 5-hmC were clearly present along the chromosomal arms (Figure 1A–1D); however, 5-mC displayed a distinctly strong signal at centromeric heterochromatin regions on all metaphase spreads examined (Figure 1B, n>5). Strikingly, at these same regions, 5-hmC appears completely depleted from 5-mC-enriched pericentromeric regions (Figure 1A, 1E–1H). Given both the defined epigenetic architecture and distinct sequence content of relatively stable centromeric heterochromatic regions, these results may suggest an association of 5-hmC with more epigenetically dynamic loci, such as those throughout chromosome arms, and perhaps exclusion from more epigenetically stable heterochromatin, such as that present in metaphase centromeres.

### Genomic features associated with 5-hmC in human embryonic stem cells

**Figure 2:**
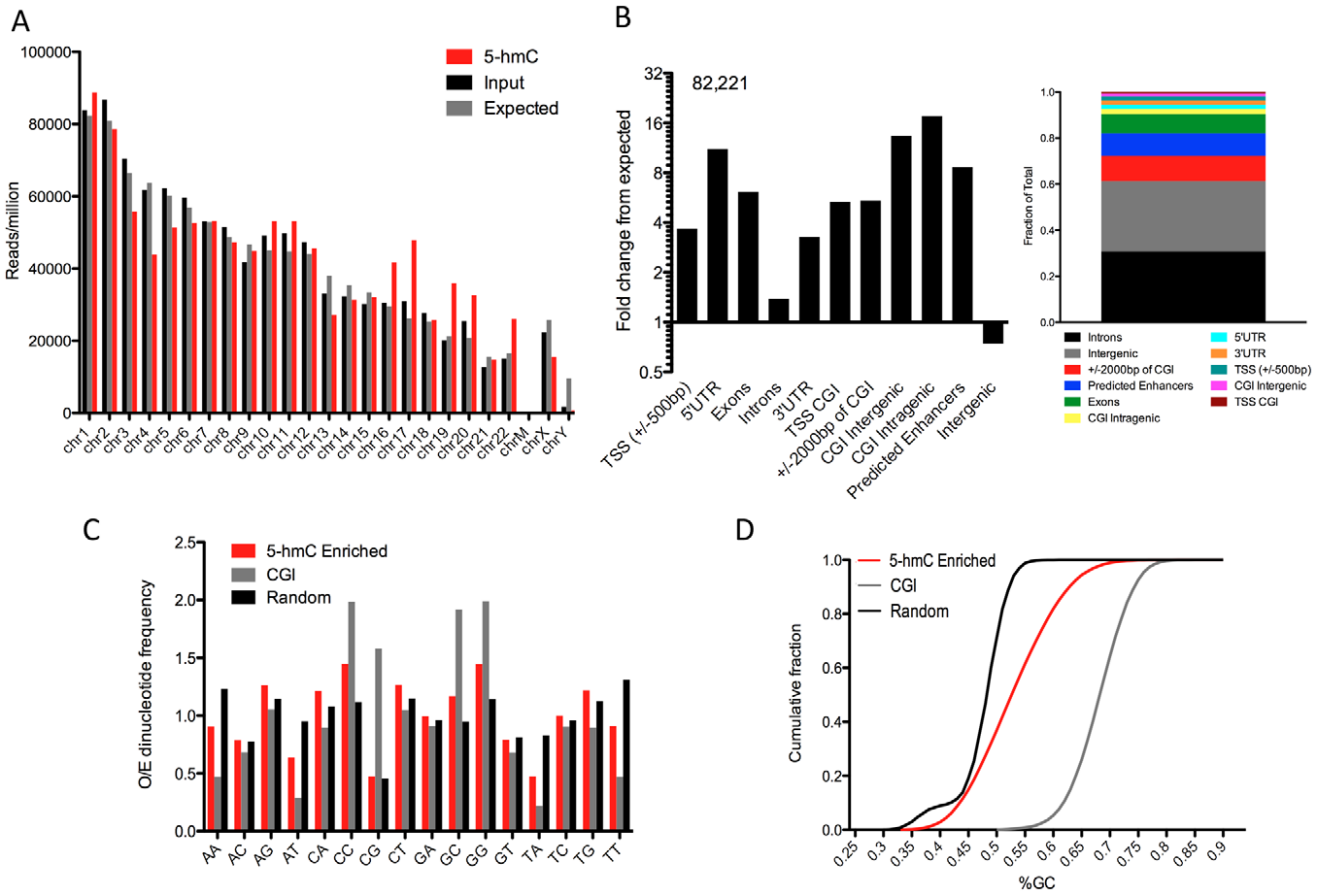
Summary of genome-wide distribution of 5-hmC in human H1 ES cells. A. Chromosome-wide distributions of 5-hmC-enriched and -unenriched input genomic DNA reads (reads/million), compared to the distribution expected by chance if reads were randomly distributed amongst chromosomes (10^6^/hg18 length X chromosome length, with expected values divided by 2 for chromosome X). B. Association between 82,221 5-hmC enriched regions (p-value threshold of 1e-8) and annotated genomic features (obtained through UCSC Tables, NCBI36/hg18 and [15]). Values are represented as the fold change in percentage of peaks overlapping a defined feature over the percent expected by chance give the genomic base coverage of that feature. Shown to the right is the fraction of total peaks corresponding to each genomic feature for reference. C. Observed-to-Expected (O/E) ratios of all possible dinucleotides in 5-hmC-enriched regions (n = 82,221, p-value threshold of 1e-8), CpG Islands (NCBI36/hg18, n = 28,226), and randomly selected genomic regions (n>100,000). D. The cumulative fraction of genomic regions with a given GC content plotted for 5-hmC-enriched regions (n = 82,221, p-value threshold of 1e-8), CpG Islands (NCBI36/hg18, n = 28,226), and randomly selected genomic regions (n>100,000).

To further evaluate the epigenomic context of 5-hmC, we first established a genome-wide map of 5-hmC in human H1 ES cells by selectively enriching 5-hmC-containing fragments of DNA and subjecting them to high-throughput sequencing. We used a previously established approach to transfer a chemically modified glucose moiety, 6-N_3_-glucose, onto the hydroxyl group of 5-hmC, which in turns allows cycloaddition of biotin for affinity enrichment and deep sequencing. We prepared and sequenced libraries from 5-hmC-enriched as well as unenriched DNA from the same preparation and sequenced to a depth of >10 million unique, non-duplicate reads per condition. Analyses of chromosome-wide 5-hmC densities showed that, while unenriched input genomic reads were distributed amongst chromosomes close to randomly, as expected by chance, 5-hmC exhibited enrichment or depletion on specific chromosomes (Figure 2A).

To further localize regions of 5-hmC enrichment, we identified 5-hmC peaks genome-wide. In total, we identified 82,221 regions as significantly enriched for 5-hmC (p-value threshold of 1e-8, Table S1). Association of 5-hmC-enriched regions with annotated genomic features indicated significant overrepresentation of 5-hmC within genes and depletion at intergenic regions (Figure 2B), consistent with what has been observed previously in both mouse cerebellum and mouse ES cells [9], [11]–[14]. Within genes, 5-hmC peaks were particularly enriched in exons (Figure 2B, 6.14-fold over expected based on the genomic coverage of these regions), whereas we saw much lower frequency within intronic regions (Figure 2B, 1.33-fold over expected), which is likely a result of the increased GC content within exons relative to introns. 5-hmC peaks were also significantly enriched within intragenic CpG islands (CGIs) (17.6-fold over expected) and are more frequent than expected by chance at intergenic CGIs (Figure 2B). Interestingly, we find significantly more 5-hmC peaks overlapping predicted enhancers than was expected (8.6-fold over expected, Figure 2B). These results indicate that in addition to gene body-associated regulatory roles, 5-hmC may also function within other genomic regions important for gene modulation.

We also assessed the general sequence content of these peaks, including GC content and dinucleotide frequencies. We found that the frequency of CpG dinucleotides within 5-hmC-enriched regions was no greater than randomly chosen regions of the genome and significantly lower than CGIs, whereas CA, CC, and CT dinucleotides each exhibited an O/E >1 and enrichment relative to random genomic locations (Figure 2C). Furthermore, GC content as a whole was significantly reduced compared with CGIs, and slightly increased relative to random genomic loci (Figure 2D). These data suggest that 5-hmC-enriched loci occur most often in regions of the genome with moderate GC content and that it occurs less frequently within a high density of CpGs.

### Genome-wide association among 5-hmC, 5-mC, and 11 histone modifications

**Figure 3:**
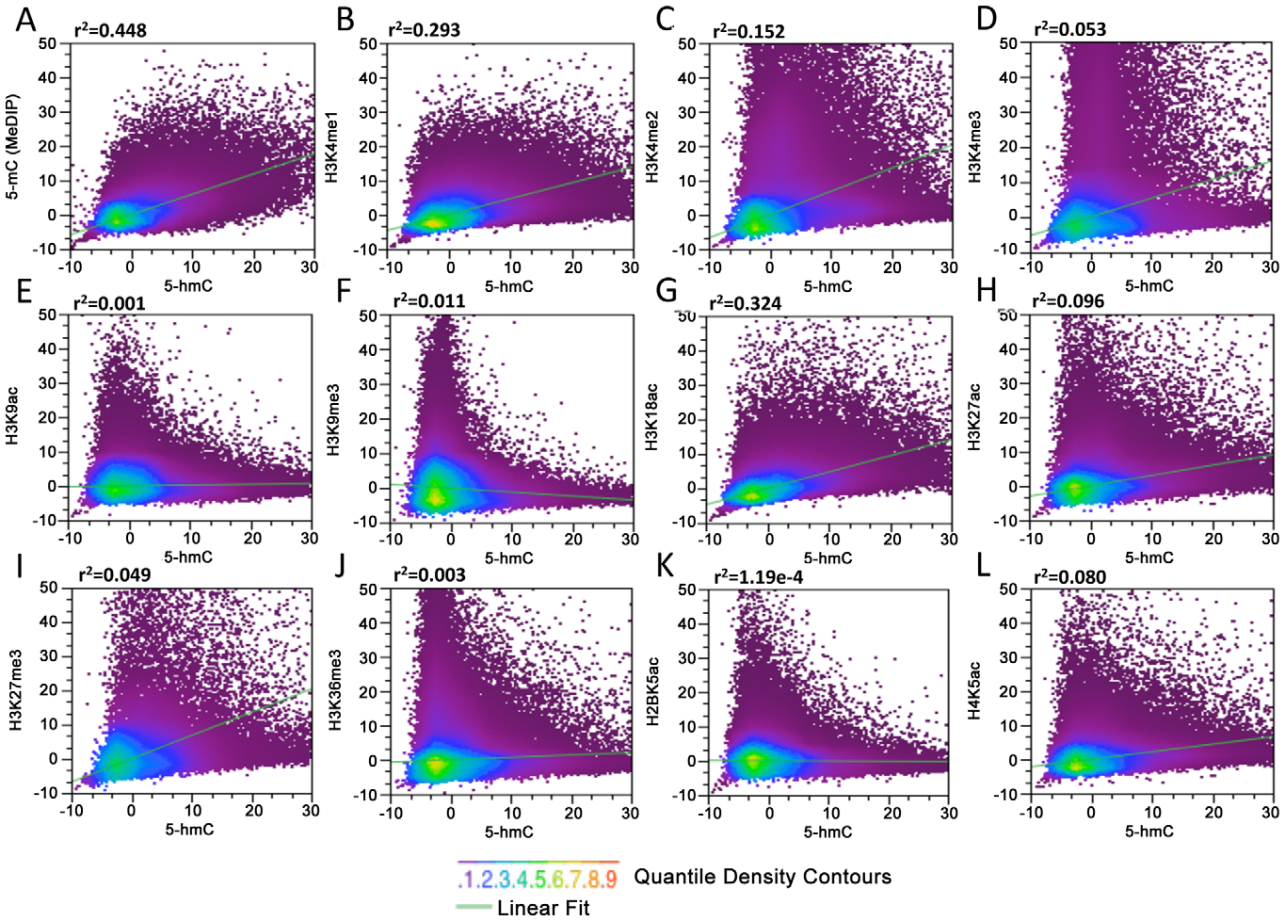
Genome-wide correlation among 5-hmC, 5-mC, and 11 distinct histone modifications in human H1 ES cells. (A–L) MeDIP and ChIP-Seq data for 11 diverse histone modifications and input from the same experiments were obtained from the previous publication and GSM456941 [15], binned at 10 kb, and normalized to the total number of aligned reads in millions. MeDIP and histone-specific values were then input-normalized (IP-Input). Data from 5-hmC-enriched and -unenriched input DNA were binned and normalized identically. Input-normalized 5-hmC signals were then plotted against histone-input normalized values to obtain correlations (r^2^).

In order to determine the specific chromatin contexts associated with 5-hmC in human embryonic stem cells, we obtained sequence data derived from immunoprecipitation of 5-mC (MeDIP) (GSM456941) and 11 diverse histone modifications in H1 hES cells [15]. MeDIP, histone-ChIP, and unenriched input reads derived from the same experiments were binned genome-wide at 1, 5, and 10 kb. MeDIP and histone-specific signals were normalized to input values (ChIP-Input). 5-hmC-enriched reads were binned genome-wide using identical parameters. Input-normalized 5-hmC signals were then subsequently correlated with input-normalized histone modification and 5-mC MeDIP values within the same genomic bin for all bins genome-wide in order to generalize the relative correlation between 5-hmC, 5-mC, and diverse histone modifications (Figure 3). We found that data binned at various sized intervals exhibited generally similar patterns on a genomic scale when comparing the relative correlations between 5-hmC and the various histone modifications tested.

We find that in general, on a genomic scale, 5-hmC and 5-mC detected by MeDIP correlate better than any histone-specific mark tested (Figure 3A, r^2^ = 0.448), consistent with the fact that 5-hmC is derived from 5-mC and with previous reports showing a significant amount of overlap between the two marks in mouse ES cell genomes [9], [11], [13]. Although it is difficult to assess the ratio of 5-mC:5-hmC from genome-wide bisulfite sequencing data (Methyl-Seq), we also determined the association between 5-hmC and 5-mC+5-hmC detected by Methyl-Seq (Figure S2). Within the CG context, regions with higher 5-hmC also tend to have a higher percentage of 5-mC+5-hmC, as would be expected. However, there are also a large number of regions with a high percentage of 5-mC+5-hmC that contain very low levels of 5-hmC and are therefore presumably dominated by 5-mC (Figure S2A). These results are again consistent with the notion that 5-hmC is derived from 5-mC. We also compared 5-hmC signals to 5-mC+5-hmC within the non-CpG context, which occurs in human ES cells more frequently than in differentiated cell types [16]. 5-hmC has been reported to occur within non-CpG contexts in mouse ES cells as well [11]. Our analyses indicate that regions containing high levels of 5-hmC tend to harbor less non-CpG methylation (Figure S2B and S2C). However, due to the low percentage of both CHG and CHH methylation throughout the genome, it is difficult to resolve the extent to which 5-hmC may occur at non-CpG sites and analyses do not exclude the possibility that 5-hmC occurs within a non-CpG context in human ES cells. Further resolution of single base pair 5-hmC will be required to conclusively establish the sequence contexts of hydroxymethylated cytosines.

Correlations between 5-hmC and the 11 histone modifications tested were largely, with a few notable exceptions, in agreement with the previously observed associations between histone modifications and the percentage of overall DNA methylation (5-mC+5-hmC) assessed by Methyl-Seq [15]. Consistent with the correlations between Methyl-Seq and histone modifications, we find a relatively strong association between 5-hmC and H3K4me1 (r^2^ = 0.293) and H3K4me2 (r^2^ = 0.152) compared with H3K4me3 (r^2^ = 0.0518) (Figure 3B–3D). The relatively strong correlations between 5-hmC, H3K4me1, and H3K4me2 compared to H3K4me3 are also consistent with earlier observations showing enrichment of 5-hmC within active gene bodies, but depletion at TSSs. We also saw a relatively strong correlation between H3K18ac, a mark that directly regulated CBP/p300 enhancer complexes with transcriptional activation [17], [18], and 5-hmC (Figure 3G, r^2^ = 0.324). A significantly smaller albeit moderate correlation was found between 5-hmC and H3K27ac, H3K27me3 (with H3K27ac > H3K27me3), and H4K5ac (Figure 3H, 3I and 3L). Both H3K9ac and H3K9me3 exhibited relatively low levels of correlation with 5-hmC (Figure 3E and 3F). Surprisingly, we see a relatively weak correlation between 5-hmC and H3K36me3 (Figure 3J). H3K36me3 is known to correlate well with gene expression levels and has been linked to transcriptional elongation in hES cells [19], but is largely absent from TSSs. H3K36me3 is also one of the few histone marks for which there is a strong correlation with methylated DNA, as detected by bisulfite sequencing [15]. These results suggest the possible enrichment of H3K36me3 or 5-hmC on distinct groups of gene bodies in hES cells, which could depend on the level of gene expression.

Together, the correlations between 5-hmC, 5-mC, and the 11 specific histone modifications tested indicate that, in addition to being generally associated with more euchromatic accessible chromatin, 5-hmC may be linked to diverse gene regulatory elements and transcriptional regulatory processes in human ES cells.

### Expression level–dependent distribution of 5-hmC at promoter-proximal regions

**Figure 4:**
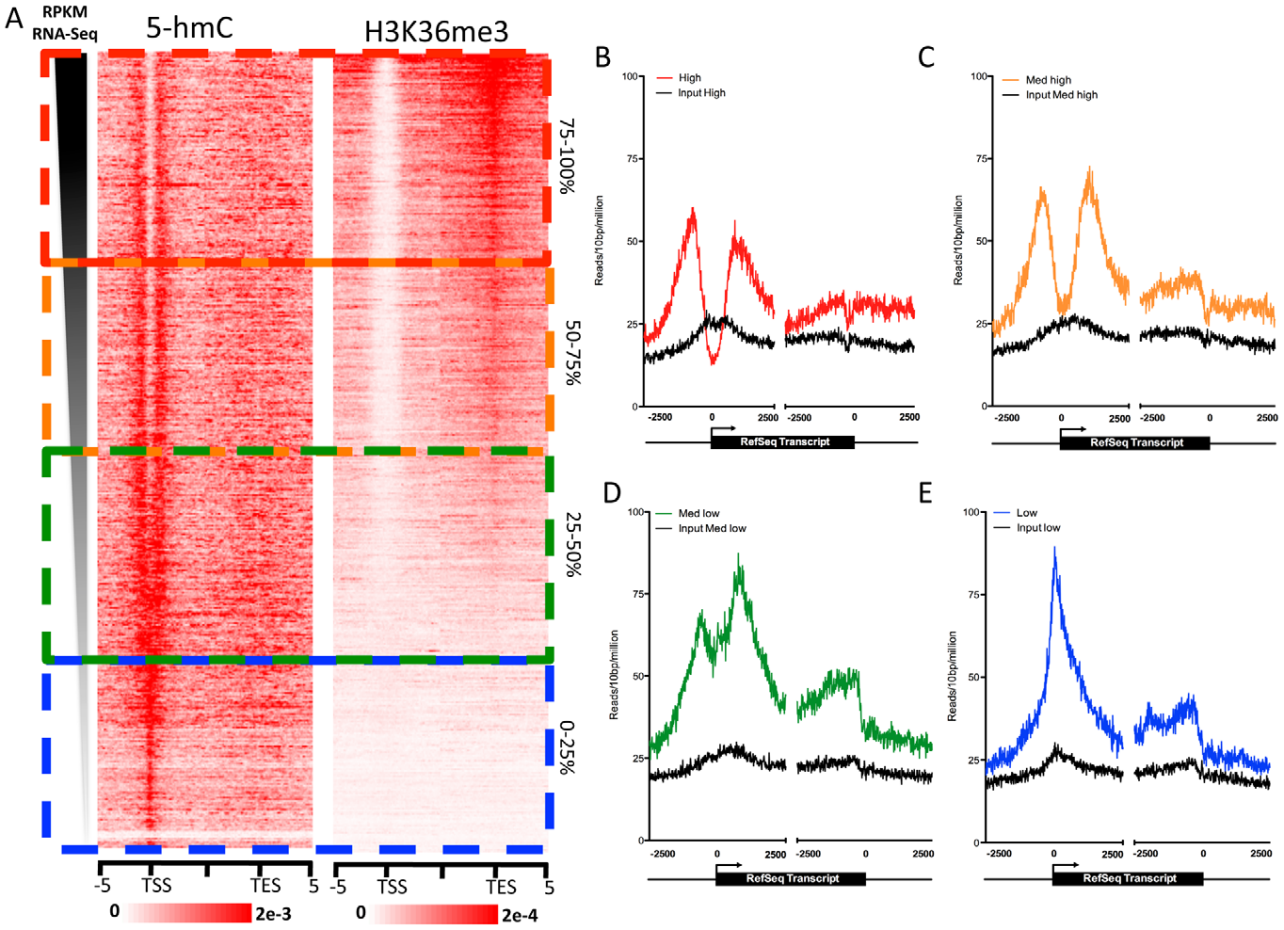
Gene expression level-dependent 5-hmC and H3K36me3 distributions in human ES cells. A. Gene expression values (RPKM) obtained from [16] were ranked in descending order, and 5-hmC and H3K36me3 read densities were measured in 100-bp bins ±5 kb of the TSS or TES. Values were normalized to the total number of aligned reads in millions. The scale of 5-hmC and H3K36me3 signals are indicated at the below each respective heatmap. B. 5-hmC and input read densities at the top 25% of genes based on RPKM expression level. C. 5-hmC and input read densities among genes expressed within the 25–50% range of all genes based on RPKM expression level. D. 5-hmC and input read densities among genes expressed within the 50–75% range of all genes based on RPKM expression level. E. 5-hmC and input read densities at the bottom 25% of genes based on RPKM expression level. For B–E, reads were summed in 10-bp windows 2.5 kb upstream and downstream of TSS and TES.

Both cytogenetic localization of 5-hmC and genome-wide correlations with 11 diverse histone modifications indicate links between 5-hmC, more accessible euchromatic chromatin, and gene regulation. To test the dependence of gene-associated 5-hmC distributions on expression levels in human ES cells, we measured 5-hmC signals at genes with varying expression as measured by RNA-Seq RPKM [16]. Overall, 5-hmC displays a strong promoter-proximal bias in hES cells, while also being enriched within gene bodies, albeit to a lesser degree relative to the TSS (Figure 4A–4E). Interestingly, we observed a distinct forking in the 5-hmC distribution around the TSS as expression levels rose, ultimately transitioning to a bimodal distribution at more highly expressed genes compared with genes expressed at lower levels (Figure 4A–4E). However, the correlation between 5-hmC and both TSSs and gene bodies is not strictly linear. 5-hmC tends to be higher, both within the gene body and at the TSS, at genes expressed within the 25–75% range of all genes based on RNA-Seq RPKM (Figure 4C and 4D), compared to the top 25% of expressed genes (Figure 4A). Meanwhile, at genes within the bottom 25%, 5-hmC is mainly enriched directly over the TSS and only moderately enriched within the gene body. Thus, at genes exhibiting lower expression, 5-hmC is present directly at the TSS (Figure 4E), whereas genes with intermediate expression display higher gene body 5-hmC and a distinct bimodal distribution (Figure 4A) at the TSS. At the most highly expressed genes, 5-hmC exhibits a similar distribution to that seen on intermediately expressed genes, but overall lower levels at both the TSS and gene body. These results are consistent with the observed dual function of 5-hmC in mouse ES cells, where the Polycomb complex PRC2 may act in combination with Tet1 to influence the distribution of 5-hmC at repressed genes, while at more highly expressed genes the presence of Tet1, without PRC2, results in loss of 5-hmC at the TSS and establishment of a bimodal distribution [9], [10].

To further explore the enrichment of 5-hmC at gene bodies with intermediate levels of expression, we directly compared the distribution of 5-hmC to that of H3K36me3 in and around genes ranked by expression level (Figure 4A). H3K36me3 is an intragenically enriched histone modification that also correlates well with gene expression levels [19]. We found that genes with the highest intragenic 5-hmC also had relatively low intragenic H3K36me3 (Figure 4A), consistent with the relatively low genome-wide correlations between binned 5-hmC and H3K36me3 (Figure 3J). The same genes were also those expressed at intermediate levels (25–75% range based on RNA-Seq RPKM). At the top 25% of expressed genes, H3K36me3 is highly enriched within gene bodies and transcription end sites (TES), while 5-hmC tends to be lower at both TSSs and gene bodies compared to genes expressed at intermediate levels (Figure 4A). These data suggest a complex relationship between 5-hmC, H3K36me3, and gene expression levels in human ES cells. One possible explanation could be that 5-hmC functions to temper transcription at the genes that are not fully committed to a constitutive expression state. At genes expressed at the lowest levels, 5-hmC may play a role at the TSS to represses full-length transcription, while still maintaining the transcriptional potential of the marked genes. Such a role is consistent with the previously reported interaction between TSS 5-hmC and repression by Polycomb group complexes, which repress many developmentally regulated genes in ES cells [9], [10]. At the genes with intermediate expression levels, 5-hmC may temper expression at both TSS and gene body. At genes with the highest expression, TSS- and gene body-associated 5-hmC may be, at least in part, replaced by H3K36me3 to allow full transcriptional potential.

We note that such distributions of 5-hmC in ES cells is distinct from that observed in mouse brain, where 5-hmC is largely depleted from TSSs, enriched within gene bodies, and correlates well with gene expression levels (Szulwach and Jin, unpublished observations and [14]). These differences may reflect stem cell-specific and brain-specific roles for 5-hmC-mediated gene regulation. Such differences may be accounted for by the relative enrichment of Tet1 in ES cells and/or yet-to-be-identified Tet-family co-factors compared to more differentiated cell types.

### 5-hmC differentially marks promoter subtypes in hES cells

**Figure 5:**
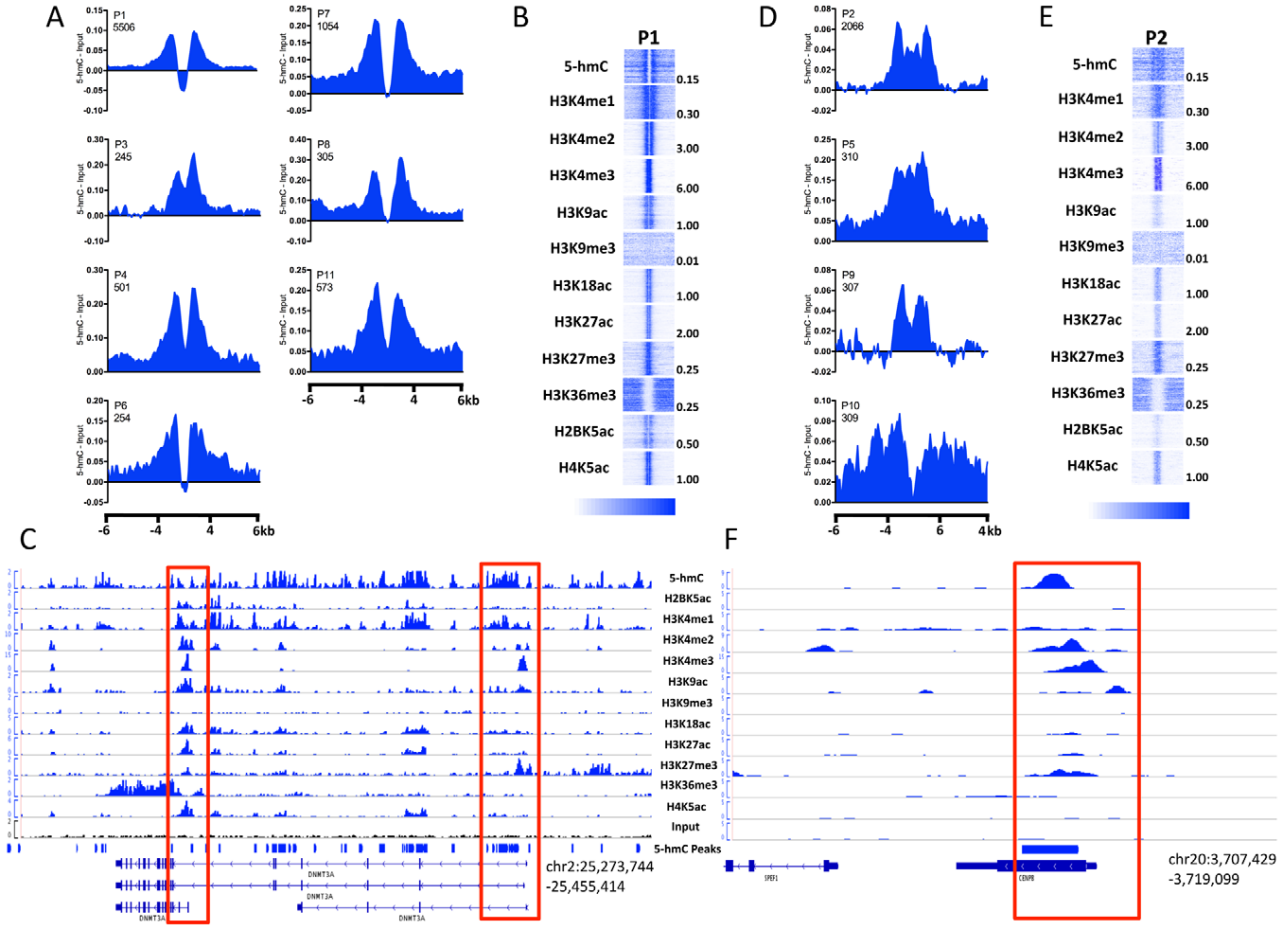
5-hmC marks distinct subtypes of promoters in human H1 ES cells. A. Distribution of 5-hmC at P1-type, bimodal promoters. 5-hmC reads were summed in 100-bp windows and the immediate 4-kb region, as well as 4 kb upstream and downstream of the immediate 4-kb region, centered on each H1 hES cell promoter type defined on the basis of their chromatin signature [15], [27]. Counts were normalized to the total number of aligned reads in millions and input reads counted and normalized in the same manner were subtracted. B. Heatmap representations of 5-hmC and 11 histone modifications at P1-type bimodal promoters. Heatmap scale is indicated below, and maximum values per mark are indicated to the right of each representative heatmap. C. Genomic view of the DNMT3A locus with read distributions for 5-hmC and 11 histone modifications exemplifying the bimodal distributions observed. Highlighted in red are the promoter regions. D. Distribution of 5-hmC at P2-type promoters. 5-hmC reads were summed in 100-bp windows in the immediate 4-kb region, as well as 4 kb upstream and downstream of the immediate 4-kb region, centered on each H1 hES cell promoter type. Counts were normalized to the total number of aligned reads in millions and input reads counted and normalized in the same manner were subtracted. E. Heatmap representations of 5-hmC and 11 histone modifications at P2-type bimodal promoters. Heatmap scale is indicated below, and maximum values per mark are indicated to the right of each representative heatmap. F. Genomic view of the CENPB locus with read distributions for 5-hmC and 11 histone modifications exemplifying P2-type promoters. Highlighted in red is the promoter region.

In promoter-proximal regions of embryonic stem cells, 5-hmC exhibits a TSS-associated bias that is dependent on gene expression level (Figure 4). To further understand the relevance of this bias in terms of chromatin context, we examined the distribution of 5-hmC around 18 distinct promoter subtypes defined on the basis of their chromatin signatures [15]. Among 11 promoter subtypes with significant enrichment of the histone modifications tested in H1 hES cells, we found that 5-hmC distributions within the same regions could be classified into two groups. The first group reflected the distribution of 5-hmC at more highly expressed genes, with 5-hmC displaying a marked depletion directly over the TSS and a bimodal distribution around the TSS (Figure 5A, 5B). This distribution corresponded to a strong H3K4me3 signal, consistent with an inverse correlation between 5-hmC and H3K4me3 (Figure 5A–5C). Flanking the region of depletion were two peaks of 5-hmC, which overlapped with regions of H4K4me1 and H3K4me2 enrichment. A clear example of this could be seen at the well-characterized promoters of the DNMT3A locus, itself a highly expressed gene in ES cells (Figure 5C). The bimodal distribution of 5-hmC, H4K4me1, and H3K4me2 around TSSs might reflect paused promoters, at which divergent RNAPII is known to display pausing, and could suggest an influence of 5-hmC on transcription pausing at such promoters in hES cells.

The second group of promoters displayed lower 5-hmC signal overall, but a more even distribution over the promoter regions, without a distinct region of depletion (Figure 5D–5F), and reflected the distribution of 5-hmC at genes expressed at intermediate or low levels (Figure 4). Again, the distribution of 5-hmC correlated well with the presence of H3K4me1 and H3K4me2, while H3K4me3 was also present (Figure 5E and 5F). We also noted that this group of promoters displayed an overall weaker signal in each histone modification tested, relative to promoters exhibiting bimodal distributions of both 5-hmC and various histone modifications (Figure 5B and 5E), which likely represents the expression status of this group of genes.

Assessment of 5-hmC at an additional seven promoter types, which displayed low levels of modified histone enrichment in H1 hES cells, also displayed low levels of 5-hmC (Figure S3) and less distinct distribution patterns, consistent with a link between defined histone modifications and 5-hmC at TSSs.

### Enrichment of 5-hmC at enhancers in hES cells

**Figure 6:**
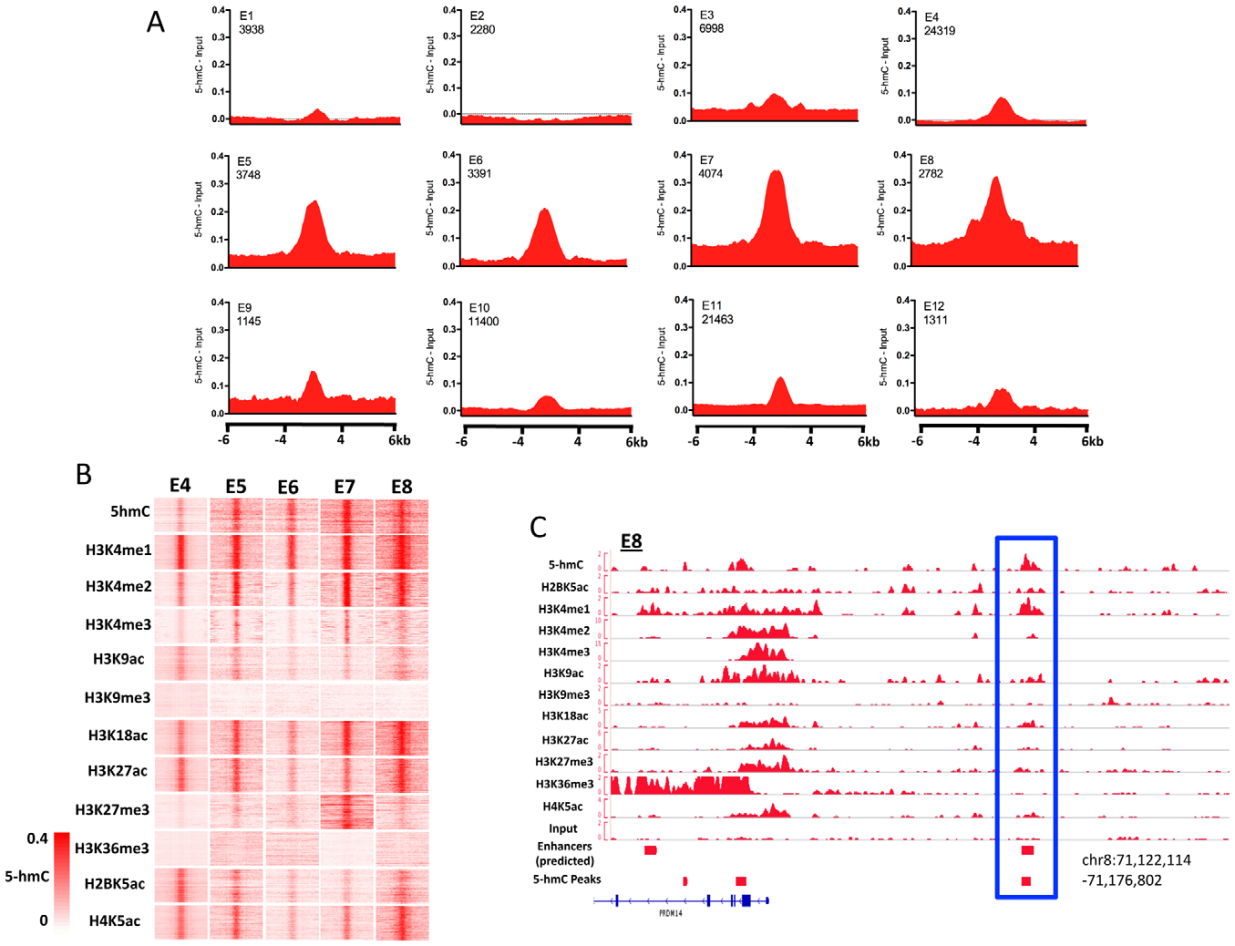
Enrichment of 5-hmC at specific enhancers in human H1 ES cells. A. 5-hmC reads were summed in 100-bp windows in the immediate 4-kb region, as well as 4 kb upstream and downstream of the immediate 4-kb region, centered on each of 12 predicted H1 hES cell enhancers defined on the basis of their chromatin signature [15], [27], [28]. Counts were normalized to the total number of aligned reads in millions and input reads counted and normalized in the same manner were subtracted [15], [27], [28]. B. Heatmap representations of read distributions for 5-hmC and 11 histone modifications at 5 predicted enhancer subtypes found to have significant enrichment in H1 hES cells. Heatmap scale is constant for all marks and indicated in the lower left-hand corner. C. Genomic view of an E8-type enhancer overlapping a region identified as significantly enriched for 5-hmC by peak identification upstream of the PRDM14 gene.

Association of 5-hmC-enriched regions with annotated genomic features suggested that, in addition to playing important roles within gene bodies and gene proximal regions, 5-hmC might also function at distinct regulatory elements, including enhancers (Figure 2B). To address the potential role of 5-hmC at enhancers as well as the distinct chromatin contexts associated with each, we determined the distribution of 5-hmC at 12 different sets of predicted enhancers defined on the basis of chromatin signature [15]. Strikingly, we found that 5-hmC marked each of five enhancer subtypes displaying enrichment of H3K4me1, H3K18ac, H4K5ac, and H3K27ac in H1 hES cells, while enhancer subtypes exhibiting less enrichment of these marks also tended to be less enriched for 5-hmC (Figure 6A and 6B). A clear example of a 5-hmC-associated enhancer occurred upstream of the ES-specific gene PRDM14, where a 5-hmC peak was identified as directly overlapping an E8 type enhancer (Figure 6C). PRDM14 has been reported as an integral factor contributing to pluripotency via interactions with the core transcriptional circuitry in ES cells [20], [21]. This may suggest a functional role for 5-hmC, in combination with at least H3K4me1, at this upstream enhancer in maintaining expression of PRDM14 and contributing to the pluripotency of human ES cells. In combination with the general enrichment of 5-hmC peaks at predicted hES cell enhancers (Figure 2B), these data demonstrate distinct marking of ES cell enhancers with 5-hmC and defined chromatin signatures.

### 5-hmC at defined ChIP-rich regions

We further tested the distribution of 5-hmC around a set of 12 ChIP-rich regions that were previously identified as exhibiting enrichment of specific histone modifications, but that lay outside of defined promoters or predicted enhancer regions (Figure S4) [15]. In general, 5-hmC signals were significantly lower at such regions, and few patterns were apparent. However, we did find that ChIP-rich regions with H3K36me3 displayed markedly lower levels of 5-hmC and that regions enriched for K3K9me3 actually exhibited depletion of 5-hmC (Figure S2), consistent with the lower genome-wide correlations we found between 5-hmC and these two histone modifications (Figure 4E and 4I).

### 5-hmC at pluripotency-associated transcription factor binding sites

**Figure 7:**
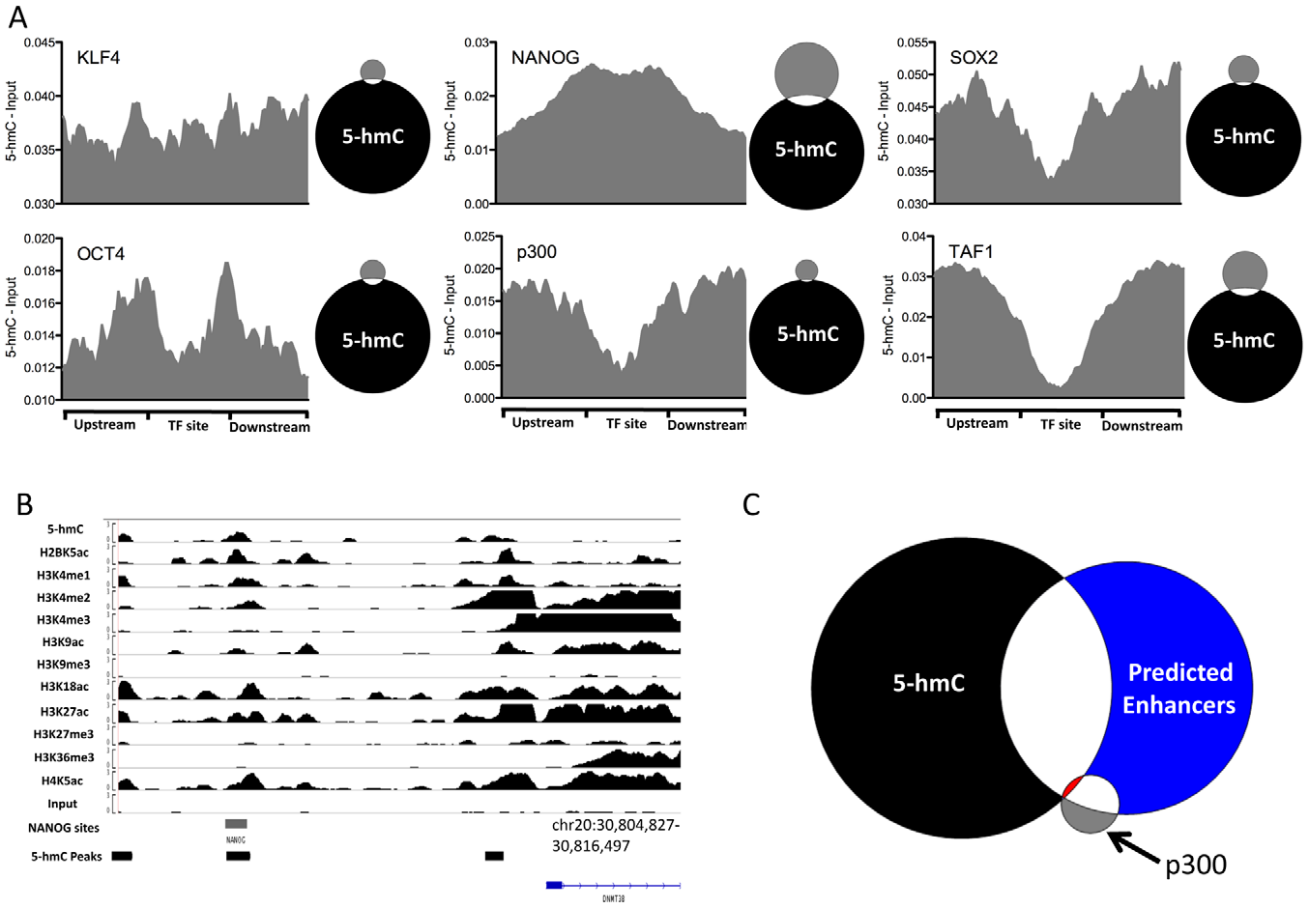
5-hmC at pluripotency-associated core transcription factor binding sites in human H1 ES cells. A. 5-hmC read distributions at KLF4 (n = 3,795), OCT4 (n = 3,890), SOX2 (n = 5,684), NANOG (n = 25,076), p300 (n = 3,094), and TAF1 (n = 12,363) binding sites in H1 hES cells. 5-hmC reads were counted in 40 equally portions within, upstream, and downstream of each binding site and values were normalized to the total number of aligned reads. Input reads counted and normalized in the same manner were then subtracted. Shown to the right of each plot is a scaled Venn diagram of the number of transcription factor binding sites (in gray) directly overlapping 5-hmC enriched regions (in black, 82,221) for reference. Overlaps are defined as ≥1 bp. B. Genomic view of 5-hmC coverage and 11 histone modifications at the DNMT3B locus, showing overlap of a 5-hmC peak, NANOG site, and the associated histone modifications. C. Scaled Venn diagram depicting the overlap between 5-hmC enriched regions (black, 82,221), p300 binding sites (gray, 3,094), and predicted enhancers reported in [15] (blue, 58,023). Shown in red is the overlap of all three (25 regions). Overlaps are defined as ≥1 bp.

DNA methylation has been implicated in regulating transcription factor binding dynamics and has been found to differentially mark sites of core pluripotency-associated transcription factors in ES cells [16]. We therefore asked whether or not 5-hmC marked sites bound by six transcription factors mapped genome-wide in H1 hES cells, including the pluripotency-associated transcription factors NANOG, OCT4, and SOX2, as well as more general factors, such as p300 and TAF1 (Figure 7). At sites of all types we could detect a slight enrichment of 5-hmC and direct overlap between subsets of 5-hmC peaks and transcription factor binding sites, consistent with previous observations in mouse ES cells detecting 5-hmC at transcription factor binding sites [9], [11], [13]. However, signals varied across factors. Among pluripotency-related factors, we find distinct marking and enrichment of 5-hmC at of only NANOG sites (Figure 7A). An example of 5-hmC enrichment at a NANOG binding site was seen directly upstream of DNMT3B (Figure 7B), a gene expressed a high levels in ES cells. Consistent with a lack of 5-hmC at many TSSs, we also observe depletion of 5-hmC at TAF1 interaction sites (Figure 7A).

Although we observed good correlation between histone modifications demarcating enhancers and enrichment of 5-hmC at specific subtypes of enhancers defined by chromatin signature, we did not observe distinct 5-hmC marking at p300 sites (Figure 7A). We further addressed this by asking what the overlap was between the 82,221 identified 5-hmC enriched regions (Table S1), predicted enhancers [15] and p300 sites [16]. As expected a large proportion of p300 sites (1795 of 3094, 58%) overlap predicted enhancers (Figure 7C). However, the fraction of predicted enhancers explained by p300 binding remained quite low (1,795 of 58,023, 3.1%), suggesting a significant amount of enhancer regulation by p300 independent mechanisms. Interestingly, we find that while only a small fraction of p300 sites (166 of 3094, 5.4%) overlap 5-hmC enriched regions, a significant percentage of predicted enhancers (19,973 of 58,023, 34.4%) overlap with 5-hmC enriched regions (Figure 7C). Furthermore, sites that were enriched for 5-hmC, bound by p300, and predicted as enhancers were quite rare, occurring only 25 times. These data suggest that significant portion of predicted enhancers are also enriched in 5-hmC, but lack p300 binding, and may indicate a role for 5-hmC in regulating p300 independent enhancers.

Together these results indicate that 5-hmC may also influence the chromatin states at protein-DNA interaction sites, thereby modulating the function of key transcription factors and diverse enhancer subtypes.

## Discussion

Recent studies have shown that Tet family proteins can catalyze 5-methylcytosince (5-mC) conversion to 5-hydroxymethylcytosine (5-hmC) and play important roles in self-renewal and cell lineage specification in embryonic stem (ES) cells [4]–[6], [11], [22]. These findings suggest a potential role for 5-hmC-mediated epigenetic regulation in modulating the pluripotency of ES cells. To unveil this new regulatory paradigm in human ES cells, here we used a selective 5-hmC chemical labeling approach coupled with affinity purification and deep sequencing that we developed before to establish the genome-wide distribution of 5-hmC in human ES cells. Integration of 5-hmC distributions with genome-wide histone profiles led us to identify the pluripotency-linked chromatin contexts associated with 5-hmC. Through association with genomic features defined on the basis of chromatin signatures, we find 5-hmC-mediated marking of not only specific promoters and gene bodies, but also distinct enhancer subtypes, including those marked with H3K4me1 and H3K27Ac. Lastly, we find 5-hmC is associated with the binding sites of specific core pluripotency transcription factors and a lack of 5-hmC at others. Our results suggest that 5-hmC is an important epigenetic modification associated with the pluripotent state that could play role(s) in a subset of promoters and enhancers with defined chromatin signatures in ES cells.

By correlating genome-wide distributions of 5-hmC with those of 11 diverse histone marks, we found that 5-hmC displayed relatively strong correlations with H3K4me1 and H3K4me2 versus H3K4me3, which, as expected, is consistent with previous correlations between DNA methylation detected by Methyl-Seq and histone modifications [15]. 5-hmC also exhibited a strong correlation with H3K18ac, a mark regulated by CBP/p300 at enhancers that is associated with transcriptional activation. We also found more modest correlations with H3K27ac, H3K27me3, and H4K5ac, and very low correlations with H3K9ac and H3K9me3. However, our data suggested that 5-hmC was not strongly correlated with H3K36me3, a histone modification previously linked to DNA methylation detected by Methyl-Seq. This intriguing difference suggested differential marking of gene bodies by 5-hmC and H3K36me3 in pluripotent cells. Direct comparisons of genic 5-hmC and H3K36me3 indeed revealed that genes with the highest levels of TSS and gene body 5-hmC tend to exhibit intermediate levels of expression and harbor less intragenic H3K36me3, compared to genes with the highest levels of expression. Although a number of intriguing explanations might account for these observations, one possibility is that 5-hmC may function to temper transcription at both the TSS and gene body of intermediately expressed genes, while maintaining their potential to be more fully expressed when needed. Upon full activation, 5-hmC may be at least partially removed as the transcriptional unit acquires H3K36me3 and commits to a more fully active state. Restriction of 5-hmC at the TSS of repressed genes and its presence at both TSSs and gene bodies of intermediately expressed genes may also indicate distinct regulation of 5-hmC at these locations. At TSSs of genes that are repressed or expressed at low levels, Polycomb group complex, PRC2, may interact with 5-hmC to repress but maintain the potential for expression of targeted genes, as has been previously suggested [9], [10]. However, such distributions are distinct from those observed in mouse cerebellum [14], where 5-hmC is significantly enriched compared to ES cells, largely absent from TSSs, and high within gene-bodies, positively correlating gene-expression. Thus, distinction of mechanisms differentially influencing the state and regulation of 5-hmC within genes bodies in the context of gene expression outcomes will be important towards understanding the role of 5-hmC in both brain and ES cells.

Our genome-wide analyses of 5-hmC also revealed a general promoter-proximal bias of 5-hmC around RefSeq transcripts in human ES cells, which is consistent with the recently published work on mapping 5-hmC in mouse ES cells [9], [11]–[13]. This TSS-associated bias was also dependent on gene expression levels, with 5-hmC transitioning from a position directly over the TSS at repressed genes to a bimodal distribution at more highly expressed genes, likely reflecting the observed dual function of 5-hmC in mouse ES cells [9]–[13], although this correlation was not strictly linear. Interestingly, we find that the bimodal distribution of 5-hmC is also strongly correlated with the distributions of H3K4me1 and H3K4me2, but inversely correlated with H3K4me3. The bimodal distribution of 5-hmC, H4K4me1, and H3K4me2 around TSSs might reflect the establishment of divergent paused RNAPII, which is known to play a critical regulatory role at developmentally regulated transcripts in ES cells [23], [24]. This could thereby point to an influence of 5-hmC on transcription pausing at such promoters in hES cells. We also noted that such a promoter-proximal bias of 5-hmC in ES cells is distinct from that observed in mouse brain, where 5-hmC is largely depleted from TSSs and enriched within gene bodies (Szulwach and Jin, unpublished observations and [14]), where it also correlates well with gene expression. This could suggest that such a bias reflects a stem cell-specific role for 5-hmC-mediated gene regulation at and around certain TSSs. Such differences may be accounted for by the enrichment of Tet1, or yet-to-be-identified co-factors of Tet1, in ES cells relative to more differentiated cell types.

Analyses of 5-hmC-enriched peaks and their correlation with enhancer-associated specific histone modifications, such as H3K4me1, H3K18ac, and H3K27ac, suggested that, in addition to being present at promoters, 5-hmC could also mark other diverse regulatory elements in the genome, such as enhancers. Interestingly, assessment of 5-hmC distributions at the predicted enhancers in H1 hES cells demonstrated the enrichment of the epigenetic mark at specific enhancer subtypes, including those enriched for K3K4me1, H3K27ac, H3K18ac, and H4K5ac. Despite a good correlation between 5-hmC and histone marks demarcating enhancers, we found that only small fraction of regions bound by p300 were also enriched for 5-hmC.

Finally, we examined the correlation of 5-hmC distributions with the genome-wide binding sites of six transcription factors that have been linked to maintaining the pluripotency of ES cells [16]. We find that 5-hmC can also mark NANOG binding sites, while being depleted at TAF1 sites. These results further suggest diverse roles for 5-hmC in regulating the accessibility of transcription factors in defined chromatin contexts, including those regulating pluripotency in ES cells.

In summary, here we present the genome-wide distribution of 5-hmC and its correlation with 11 diverse histone modifications and six transcription factors in human ES cells. By integrating genomic 5-hmC signals with maps of different histone marks, we link particular pluripotency-associated chromatin contexts with 5-hmC. Our study suggests that 5-hmC could play diverse roles in regulating specific promoters, gene bodies, and enhancers in ES cells, thereby providing a detailed epigenomic map of 5-hmC from which to study its contribution to pluripotency.

## Materials and Methods

### Human ES cell culture

H1 human ES cells were maintained on mitomycin C-treated STO cells in ES medium consisting of DMEM/F12 medium (Invitrogen) supplemented with 20% serum replacement (SR; Invitrogen), 1 mM L-glutamine (Invitrogen), 100 µM nonessential amino acids (Invitrogen), 0.1 mM ß-mercaptoethanol (Sigma), 1X Antibiotics-Antimycotic (Invitrogen), and 4 ng/mL bFGF (Invitrogen). The fully grown H1 cells were mechanically isolated and transferred into a prepared dish with fresh feeder cells. Prior to the isolation of genomic DNA, cells were treated with dispase (2 mg/ml in DMEM/F12) to detach human ES cells from feeder cells.

### Metaphase chromosome preparation and staining

Metaphase chromosomes were prepared by standard protocols as described previously [25]. The slides with hES metaphase chromosome spreads were washed with PBS for 5 min. The slides were immersed in 1N HCl and incubated at 37°C for 30 min. After HCl treatment, the slides were washed with PBS for 15 min followed by blocking with 3% goat serum/0.4 Triton X-100 in PBS for 1 h. The samples were incubated with primary antibodies at 4°C overnight. The following primary antibodies were used: rabbit anti 5-hydroxymehtylcytosine (1∶10,000, #39769, Active Motif), mouse anti-5-methylcytosine (1∶1000, Eurogentec, BI-MECY-0100). On the second day, the slides were washed with PBS and then incubated with secondary antibodies: goat anti-rabbit Alexa488 (1∶500, #A11008, Invitrogen) and goat anti-mouse Alexa568 (1∶500, #A11031, Invitrogen). The slides were counter-stained with the fluorescent nuclear dye 4′,6-diamidino-2-phenylindole (#B2261, Sigma). The slides were examined using a Zeiss AX10 microscope, and images were processed with Photoshop software. More than 5 metaphase spreads were examined for 5-mC and 5-hmC.

### Genomic DNA preparation

Genomic DNA was isolated by cell lysis in digestion buffer (100 mM Tris-HCl, pH 8.5, 5 mM EDTA, 0.2% SDS, 200 mM NaCl), Proteinase K treatment (0.667 ug/ul, 55°C overnight). The second day, an equal volume of Phenol:Chloroform:Isoamyl Alcohol (25∶24∶1 Saturated with 10 mM Tris, pH 8.0, 1 mM EDTA) (P-3803, Sigma) was added to samples, mixed completely, and centrifuged for 5 min at 14,000 rpm. The aqueous layer solution was transferred into a new Eppendorf tube and precipitated with 2 volumes 100% ethanol and 1/10 volume 3 M NaOAc. The genomic DNA was recovered and dissolved with 10 mM Tris-HCl, pH 8.0. Genomic DNA samples were further sonicated into ∼500 bp by Misonix sonicator 3000 (using microtip, 4 pulses of 27 s each, with 1 min of rest and a power output level of 2; the sonication was performed always on ice). The fragment size of sonicated DNA was verified by agarose gel electrophoresis. The DNA concentration was determined with NANO-DROP 1000 (Thermo Scientific).

### 5-hmC and 5-mC dot blot

The dot blot was performed on a Bio-Dot Apparatus (#170-6545, BIO-RAD). Briefly, the serially diluted C, 5-mC, or 5-hmC only standard DNA samples (Zymo research) were mixed with 2N NaOH and 10 mM Tris·Cl, pH 8.5, and loaded onto 6X SSC rinsed Hybond-N+ membrane (Amersham Biosciences, #RPN303B). The completely dried membrane was baked for 30 min at 80°C and then blocked with PBS containing 5% dry milk and 0.1% Triton X-100 for 1 h at room temperature. The primary rabbit anti-5-hydroxymethylcytosine antibody (1∶10,000, #39769, Active Motif) or (1∶1,000, mouse monoclonal anti-5-methylcytosine, BI-MECY-0100, Anaspec) was applied to the membrane and incubated overnight at 4°C. The second day, the membrane was rinsed with PBS and the signal was developed after incubation with HRP-conjugated secondary antibody for 30 min.

### 5-hmC–specific chemical labeling and affinity purification

5-hmC enrichment was performed using a previously described procedure with an improved selective chemical labelling method [14]. Briefly, the 5-hmC labelling reactions were performed in a 100-µL solution containing 50 mM HEPES buffer (pH 7.9), 25 mM MgCl_2_, 300 ng/µL sonicated genomic DNA (100–500 bp), 250 µM UDP-6-N_3_-Glu, and 2.25 µM wild-type β-GT. The reactions were incubated for 1 h at 37°C. After the reaction, the DNA substrates were purified via Qiagen DNA purification kit or by phenol-chloroform precipitation and reconstituted in H_2_O. The click chemistry was performed with the addition of 150 µM dibenzocyclooctyne-modified biotin into the DNA solution, and the reaction mixture was incubated for 2 h at 37°C. The DNA samples were then purified by Pierce Monomeric Avidin Kit (Thermo) following the manufacturer's recommendations. After elution, the biotin-5-N_3_-gmC-containing DNA was concentrated by 10 K Amicon Ultra-0.5 mL Centrifugal Filters (Millipore) and purified by Qiagen DNA purification kit.

### Sequencing of 5-hmC–enriched and input genomic DNA

DNA libraries were generated following the Illumina protocol for “Preparing Samples for ChIP Sequencing of DNA” (Part# 111257047 Rev. A). We used 25 ng of input genomic DNA or 5-hmC-captured DNA to initiate the protocol. DNA fragments of ∼150–300 bp were gel-purified after the adapter ligation step. PCR-amplified DNA libraries were quantified on an Agilent 2100 Bioanalyzer and diluted to 6-8 pM for cluster generation and sequencing. We performed 38-cycle single-end sequencing using Version 4 Cluster Generation and Sequencing Kits (Part #15002739 and #15005236 respectively) and Version 7.0 recipes. Image processing and sequence extraction were done using the standard Illumina Pipeline.

### Sequence alignment, binning, and peak identification

FASTQ sequence files were aligned to the Human reference (NCBI36, hg18) using Bowtie 0.12.6, retaining only unique, non-duplicate genomic matches with no more than 2 mismatches within the first 25 bp.

Unique, non-duplicate reads from non-enriched input genomic DNA and each 5-hmC-enriched sequence set were counted in 1000-, 5000-, and 10,000-bp bins genome-wide and subsequently normalized to the total number of non-duplicate reads in millions. We find that bins of varying size produce largely similar patterns genome wide and have reported values within a bin size of 10 kb within all figures. Input-normalized values were subtracted from 5-hmC-enriched values per bin to generate normalized 5-hmC signals.

Summary of sequence output: H1, 5-hmC enriched  = 10038770 non-duplicate reads, H1, Unenriched input  = 20656172 non-duplicate reads.

Chromosome-wide densities were determined as reads per chromosome divided by the total number of reads in millions. Expected values were determined by dividing 10^6^ by the total NCBI36/hg18 length, and multiplying by chromosomal length. Expected values were divided by 2 for chromosomes X and Y.

For MeDIP/histone modification correlations, unique, non-duplicate reads from non-enriched input genomic DNA and 5-hmC-enriched DNA were counted in 10,000-bp bins genome-wide and subsequently normalized to the total number of non-duplicate reads in millions. Input-normalized values were subtracted from 5-hmC/histone-enriched values per bin to generate normalized 5-hmC/histone signals. All histone ChIP-Seq data was acquired from Sequence Read Archive (SRA), accession SRP000941, [15]. MeDIP data were obtained from NCBI GEO Accession GSM456941. All histone ChIP-Seq data and MeDIP were mapped and processed with the identical parameters used for 5-hmC reads described above.

5-hmC peaks were identified using MACS [26] with the following parameters: effective genome size  = 2.7e+09; Tag size  = 38; Bandwidth  = 200; P-value cutoff  = 1.00e-08; ranges for calculating regional lambda are: peak_region, 200, 1000.

Association of 5-hmC peaks with genomic features was performed by overlapping peak locations with known genomic features obtained from UCSC Tables for NCBI36/hg18: RefSeq Whole Gene, 5′UTR, Exon, Intron, 3′UTR, +/−500 bp of RefSeq TSS, RefSeq Intergenic (complement of Whole Gene), CpG Islands (+/−2 kb of CGI, Intergenic/Intragenic/TSS based on RefSeq Whole Gene). Predicted enhancer locations were obtained from [15]. Peaks were assigned to a given genomic feature if overlapping ≥1 bp. Expected values were determined based on the percent base coverage of each defined genomic feature in NCBI36/hg18.

### Histone ChIP-Seq and chromatin signatures

All histone ChIP-Seq data were acquired from Sequence Read Archive (SRA), accession SRP000941. All histone ChIP-Seq data were mapped and processed with the identical parameters used for 5-hmC reads described above. Chromatin signatures for promoters, enhancers, and ChIP-rich regions were acquired from [15]. 5-hmC reads were counted in 100-bp bins, in the 4 kb directly surrounding identified binding sites, as well as 4 kb upstream and downstream of the immediate 4-kb region. Read counts were normalized to the total number of aligned reads in millions and input reads counted and normalized in the same manner were subsequently subtracted to determine 5-hmC enrichment.

### Pluripotency-associated transcription factor binding sites and H1 hES RNA-Seq

RNA-Seq RPKM values and transcription factor binding sites for KLF4, NANOG, OCT4, p300, SOX2, and TAF1 in H1 ES cells were described previously [16]. For correlations between 5-hmC and gene expression, 5-hmC reads were counted in 100-bp bins, in the ±5 kb directly surrounding TSSs and TESs. Read counts were normalized to the total number of aligned reads in millions. For transcription factor binding sites, 5-hmC reads were counted in 40 equally sized portions within, upstream, and downstream of the binding sites. Read counts were normalized to the total number of aligned reads in millions and input reads counted and normalized in the same manner were subtracted to determine 5-hmC enrichment.

### Methyl-seq data and analysis

Methyl cytosine counts in the CG, CHG, or CHH context were obtained directly from [16] and the percent methylation in each 10 kb bin genome-wide was determined as the weighted sum of methylated cytosine detected at each position.

## Supporting Information

Figure S1 Verification of 5-mC and 5-hmC antibody specificity. Dot blots of cytosine only (C), 5-methylcytosine only (mC), or 5-hydroxymethylcytosine only (5-hmC), control DNA (949 bp, Zymo Research) demonstrating the specificity of anti-5 mC and anti-5-hmC antibodies used on metaphase spreads.
(TIF)

Figure S2 Correlation between 5-hmC and methyl-seq CG, CHG, and CGG DNA methylation. A. 5-hmC versus percent 5-mCG detected by methyl-seq. B. 5-hmC versus percent 5-mCHG detected by methyl-seq. C. 5-hmC versus percent 5-mCHH detected by methyl-seq. For all plots 5-hmC and input reads were counted in 10 kb bins, normalized to the total number of aligned reads in millions and input values were subtracted from 5-hmC values. Methyl cytosine counts in the CG, CHG, or CHH context were obtained directly from [16] and the percent methylation in each 10 kb bin genome-wide was determined as the weighted sum of methylated cytosine detected at each position.
(TIF)

Figure S3 Distribution of 5-hmC at P12–P18 type promoters previously defined on the basis of their chromatin signature [15], [27]. 5-hmC reads were summed in 100-bp windows and the immediate 4 kb region, as well as 4 kb upstream and downstream of the immediate 4 kb region, centered on each H1 hES cell promoter type. Read counts were normalized to the total number of read in millions and input reads counted and normalized in the same manner were subtracted.
(TIF)

Figure S4 Distribution of 5-hmC at 12 ChIP-rich regions previously defined on the basis of their chromatin signature that lie outside known promoters and predicted enhancers [15], [27]. 5-hmC reads were summed in 100-bp windows and the immediate 4 kb region, as well as 4 kb upstream and downstream of the immediate 4 kb region, centered on each H1 hES cell promoter type. Read counts were normalized to the total number of read in millions and input reads counted and normalized in the same manner were subtracted. Histone marks identified as enriched at particular ChIP-rich regions are indicated to the right of the specific regions.
(TIF)

Table S1 List of 82,221 5-hmC enriched regions in H1 hES cells.
(XLSX)
