# Supplementary figures and images for: Integrating 5-Hydroxymethylcytosine into the Epigenomic Landscape of Human Embryonic Stem Cells
Source: PLoS Genet. 2011 Jun 23;7(6):e1002154. doi: 10.1371/journal.pgen.1002154 (PMC3121778; doi:10.1371/journal.pgen.1002154)

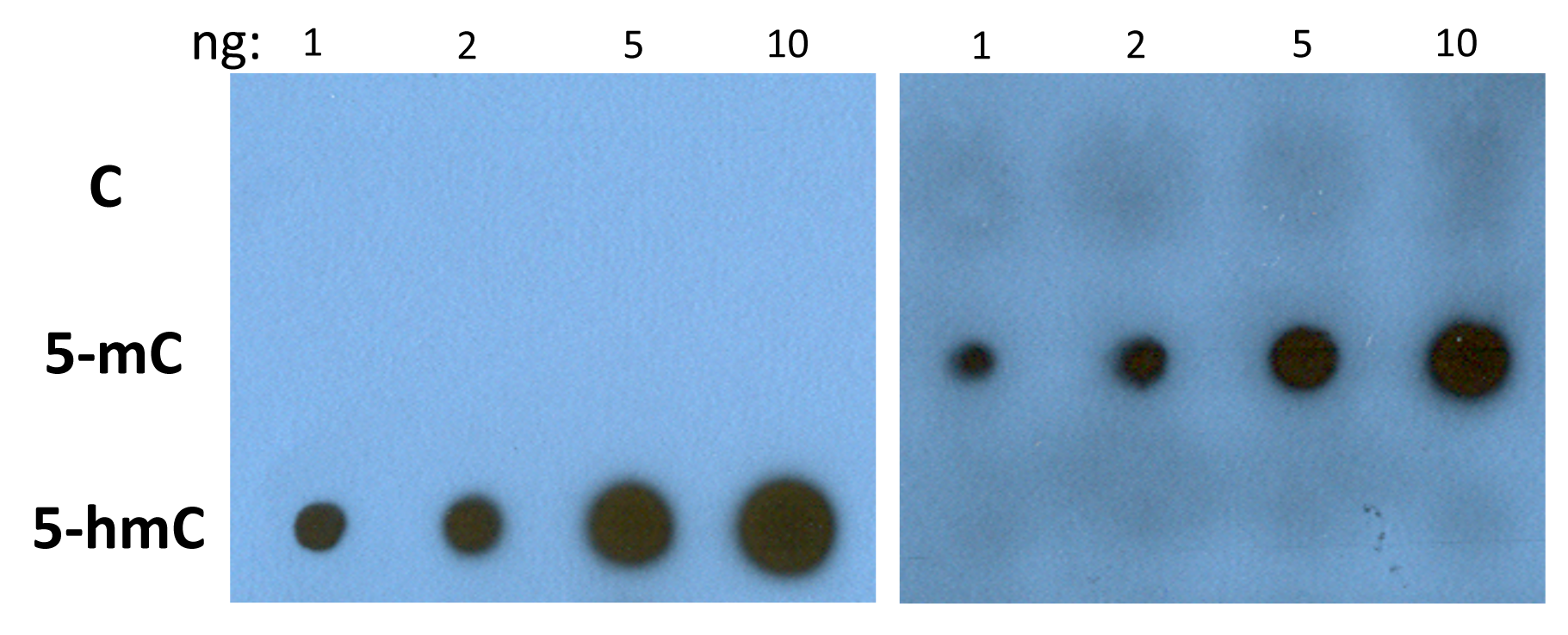

Supplement: Figure S1 — Verification of 5-mC and 5-hmC antibody specificity. Dot blots of cytosine only (C), 5-methylcytosine only (mC), or 5-hydroxymethylcytosine only (5-hmC), control DNA (949 bp, Zymo Research) demonstrating the specificity of anti-5 mC and anti-5-hmC antibodies used on metaphase spreads. (TIF) [file pgen.1002154.s001.tif]

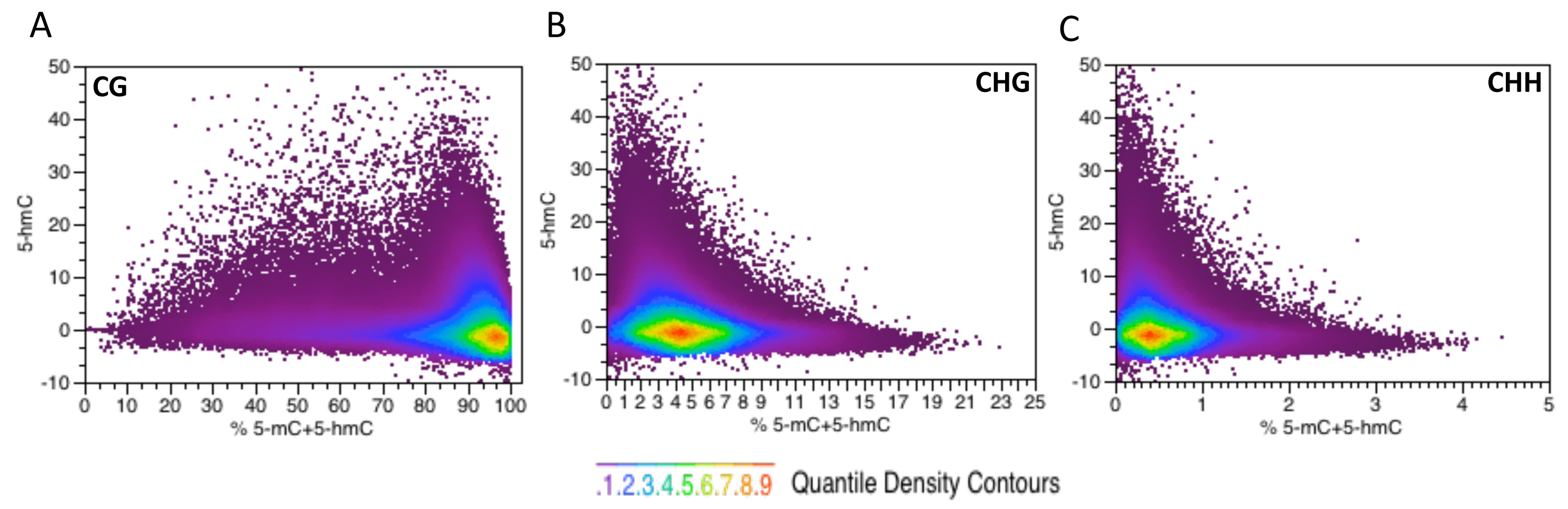

Supplement: Figure S2 — Correlation between 5-hmC and methyl-seq CG, CHG, and CGG DNA methylation. A. 5-hmC versus percent 5-mCG detected by methyl-seq. B. 5-hmC versus percent 5-mCHG detected by methyl-seq. C. 5-hmC versus percent 5-mCHH detected by methyl-seq. For all plots 5-hmC and input reads were counted in 10 kb bins, normalized to the total number of aligned reads in millions and input values were subtracted from 5-hmC values. Methyl cytosine counts in the CG, CHG, or CHH context were obtained directly from [16] and the percent methylation in each 10 kb bin genome-wide was determined as the weighted sum of methylated cytosine detected at each position. (TIF) [file pgen.1002154.s002.tif]

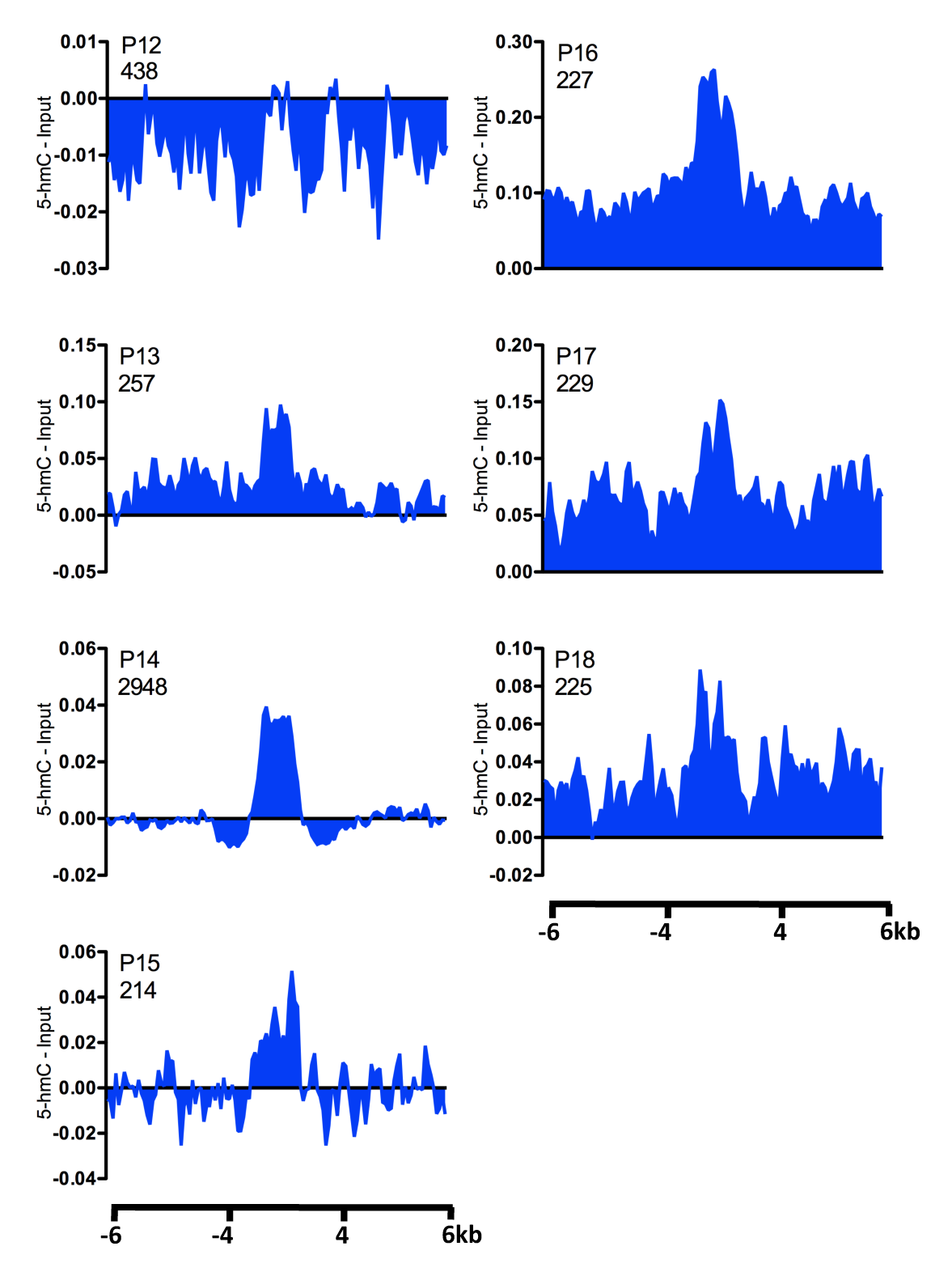

Supplement: Figure S3 — Distribution of 5-hmC at P12–P18 type promoters previously defined on the basis of their chromatin signature [15], [27]. 5-hmC reads were summed in 100-bp windows and the immediate 4 kb region, as well as 4 kb upstream and downstream of the immediate 4 kb region, centered on each H1 hES cell promoter type. Read counts were normalized to the total number of read in millions and input reads counted and normalized in the same manner were subtracted. (TIF) [file pgen.1002154.s003.tif]

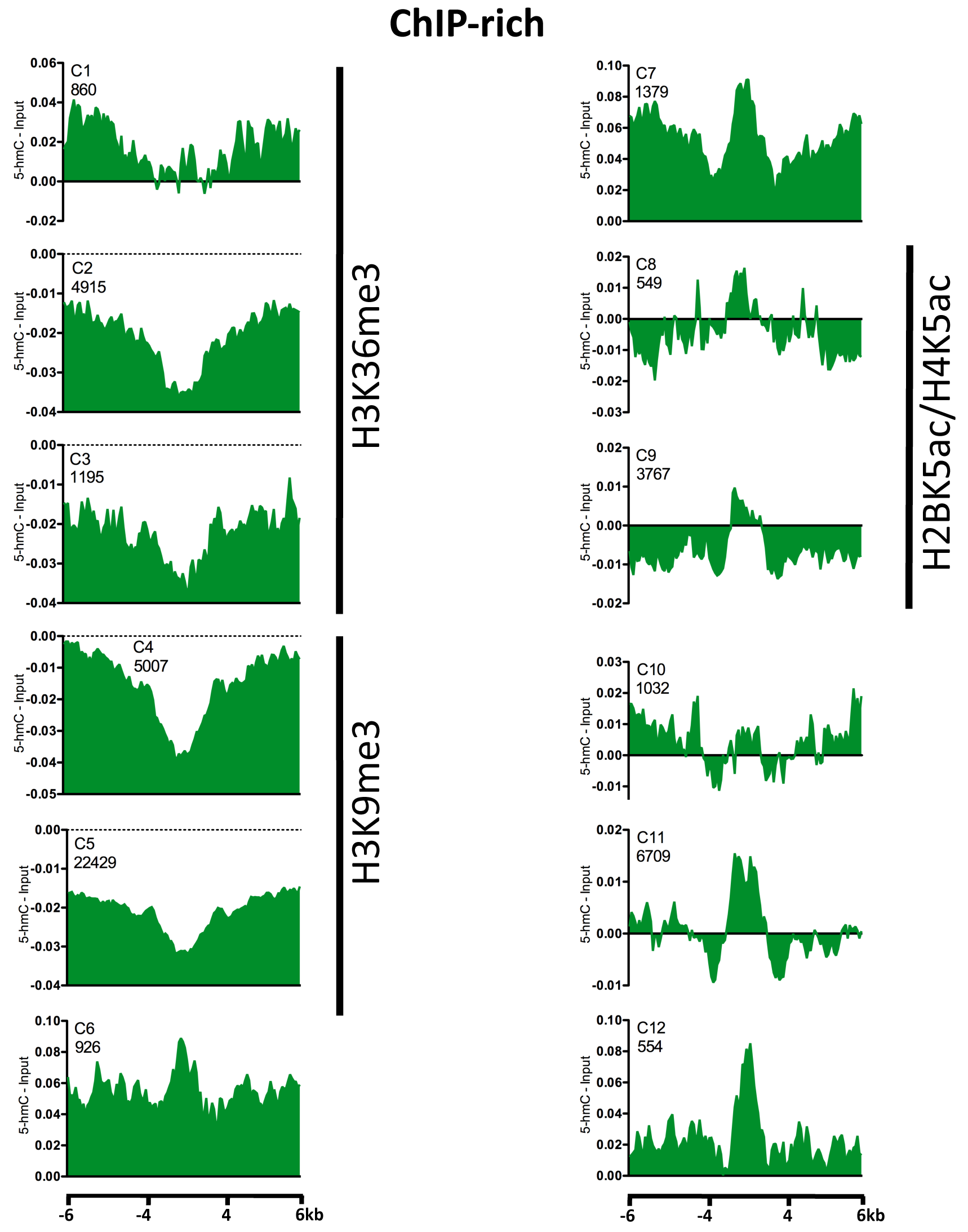

Supplement: Figure S4 — Distribution of 5-hmC at 12 ChIP-rich regions previously defined on the basis of their chromatin signature that lie outside known promoters and predicted enhancers [15], [27]. 5-hmC reads were summed in 100-bp windows and the immediate 4 kb region, as well as 4 kb upstream and downstream of the immediate 4 kb region, centered on each H1 hES cell promoter type. Read counts were normalized to the total number of read in millions and input reads counted and normalized in the same manner were subtracted. Histone marks identified as enriched at particular ChIP-rich regions are indicated to the right of the specific regions. (TIF) [file pgen.1002154.s004.tif]
